# Associations of *OPRM1*, *COMT*, and *ABCB1* Variants with Opioid Analgesic Response in Acute Renal Colic: A Candidate-Gene Study

**DOI:** 10.3390/ph19091343

**Published:** 2026-08-24

**Authors:** Sıtkı Ün, Ramazan Sabırlı, İbrahim Türkçüer, Gergana Lengerova, Martina Bozhkova, Steliyan Petrov, Aylin Köseler

**Affiliations:** 1 Department of Urology, Faculty of Medicine, Pamukkale University, Denizli 20160, Turkey; sun@pau.edu.tr; 2 Department of Emergency Medicine, Faculty of Medicine, Bakırçay University, İzmir 35665, Turkey; ramazan_sabirli@hotmail.com; 3 Department of Emergency Medicine, Faculty of Medicine, Pamukkale University, Denizli 20160, Turkey; iturkcuer@pau.edu.tr; 4 Department of Medical Microbiology and Immunology “Prof. Dr. Elissay Yanev”, Medical University of Plovdiv, 4002 Plovdiv, Bulgaria; 5 Center of Competence–Personalized Innovative Medicine, 4002 Plovdiv, Bulgaria; steliyan.petrov@mu-plovdiv.bg; 6 Research Institute, Medical University of Plovdiv, 4002 Plovdiv, Bulgaria; 7 Department of Biophysics, Faculty of Medicine, Pamukkale University, Denizli 20160, Turkey

**Keywords:** acute renal colic, pharmacogenetics, OPRM1, COMT, ABCB1, opioid analgesics, personalized medicine, pain management, genetic polymorphism

## Abstract

**Background**: Acute renal colic is a common urological emergency characterized by substantial interindividual variability in analgesic response. Pharmacogenetic variation in *OPRM1*, *COMT*, and *ABCB1* may contribute to differences in opioid efficacy and pain control. This study primarily evaluated the associations of *OPRM1* A118G (rs1799971), *COMT* Val158Met (rs4680), and *ABCB1* C3435T (rs1045642) polymorphisms with opioid analgesic response in patients with acute renal colic. As a secondary exploratory analysis, genotype and allele frequencies were compared between patients and healthy controls. **Methods**: This prospective case–control study included 150 patients with acute renal colic and 100 healthy controls. Genotyping was performed using TaqMan SNP Genotyping Assays based on real-time polymerase chain reaction. Genotype frequencies were compared between groups using dominant and recessive genetic models, and Hardy–Weinberg equilibrium was assessed. In addition, genotype–phenotype associations were evaluated using pain severity, early analgesic response, initial opioid dose, rescue analgesic requirement, and multivariable logistic regression analyses. **Results**: In the secondary exploratory case–control analysis, no statistically significant differences in genotype or allele frequencies of *OPRM1* rs1799971, *COMT* rs4680, or *ABCB1* rs1045642 were observed between patients with acute renal colic and healthy controls. Within the patient cohort, however, genotype–phenotype analyses identified differences in early analgesic outcomes. Baseline-adjusted 30 min VAS differed according to *OPRM1*, *COMT*, and *ABCB1* genotype, with the most pronounced difference observed for *ABCB1* rs1045642. Patients with the *ABCB1* TT genotype had higher adjusted 30 min VAS scores and showed a pattern of greater opioid requirement and more frequent rescue analgesia. In exploratory multivariable analysis, the *ABCB1* TT genotype was associated with higher odds of inadequate early analgesic response (adjusted OR = 2.74, 95% CI 1.18–6.37; *p* = 0.019). Given the limited number of outcome events, this adjusted association should be considered preliminary and hypothesis-generating. **Conclusions**: No significant differences in the distributions of the polymorphisms investigated were observed between patients with acute renal colic and healthy controls. Within the patient group, *ABCB1* genetic variation was associated with early opioid analgesic response, although this finding should be considered preliminary and requires confirmation in larger prospective pharmacogenetic studies before clinical implementation. Any potential future pharmacogenetic application should be considered as an adjunct to established first-line renal–colic management and specifically in patients for whom opioid therapy is clinically indicated.

## 1. Introduction

Acute renal colic, characterized by sudden and severe pain, is one of the most common urological reasons for emergency department visits. Rapid and effective pain control is a primary goal of treatment. Nonsteroidal anti-inflammatory drugs (NSAIDs) are generally recommended as first-line therapy; however, opioid analgesics are often required in patients with severe or treatment-resistant pain. Substantial interindividual variability in pain perception, analgesic efficacy, and opioid requirements cannot be fully explained by clinical factors alone, suggesting that genetic variation may also contribute to differences in analgesic response [1,2,3,4].

Pharmacogenetics investigates the contribution of genetic variation to the pharmacokinetic and pharmacodynamic effects of drugs, with the aim of improving therapeutic efficacy and reducing adverse effects as part of personalized medicine. Among the genes most extensively investigated in relation to opioid analgesic response are *OPRM1*, *COMT*, and *ABCB1*. *OPRM1* encodes the μ-opioid receptor, the principal molecular target of opioid analgesics, and the A118G (rs1799971) polymorphism has been associated with differences in opioid sensitivity and analgesic response [5,6,7]. The *COMT* Val158Met (rs4680) polymorphism influences catecholamine metabolism and has been associated with interindividual variability in pain perception and opioid requirements [8,9,10].

*ABCB1* encodes P-glycoprotein (P-gp), an ATP-dependent efflux transporter expressed at several tissue barriers, including the blood–brain barrier, where it may contribute to variability in drug disposition and central nervous system exposure to certain opioid analgesics. The synonymous *ABCB1* C3435T (rs1045642) variant has been investigated in relation to *ABCB1* expression, P-glycoprotein activity/function, drug disposition, and opioid response [11,12]. However, the reported associations have been inconsistent, and rs1045642 should not be interpreted as a variant that directly or conclusively determines P-glycoprotein function. Rather, it may represent a marker of *ABCB1*-related variability, potentially including linkage with other functional variants or haplotypes.

Recent pharmacogenetic studies have suggested that variation in these genes may be associated with opioid requirements, analgesic response, pain sensitivity, and opioid-related adverse effects [13,14,15,16,17]. Genotype-informed approaches to drug selection and dose optimization have therefore been proposed as potential strategies for improving therapeutic efficacy and reducing adverse effects [18,19,20,21]. Nevertheless, evidence supporting their routine clinical implementation remains limited, particularly in acute pain settings. The three variants investigated in the present study were selected because they represent biologically complementary mechanisms potentially contributing to variability in opioid analgesia: *OPRM1* rs1799971 represents opioid receptor-mediated pharmacodynamic variability, *COMT* rs4680 represents variability in catecholamine metabolism and pain processing, and *ABCB1* rs1045642 represents potential transporter-related variability in central opioid exposure [13,14,15,16,17,18]. Although these variants have been investigated in relation to pain perception, opioid requirements, and analgesic response in other clinical settings, evidence specifically addressing their combined relationship with opioid analgesic outcomes in acute renal colic remains limited [13,14,15,16,17]. Their associations with early pain response, opioid requirement, and rescue analgesic use in this acute clinical setting have not been adequately characterized. Our previous pharmacogenetic studies demonstrated associations between genetic variability in drug-metabolizing enzymes and analgesic response in different clinical settings, including postoperative tramadol therapy, paracetamol treatment for acute orthopedic pain, and meperidine administration in renal colic [22,23,24]. These findings provide a rationale for further investigation of pharmacogenetic variability in acute pain management.

Accordingly, the primary objective of this preliminary candidate-gene pharmacogenetic association study was to evaluate the associations of *OPRM1* A118G (rs1799971), *COMT* Val158Met (rs4680), and *ABCB1* C3435T (rs1045642) with clinically relevant opioid analgesic outcomes in patients with acute renal colic. Baseline-adjusted 30 min VAS was considered the principal measure of early analgesic response, whereas ΔVAS was evaluated as a sensitivity outcome; opioid requirement and rescue analgesic use were evaluated as additional exploratory outcomes. As a secondary exploratory objective, genotype and allele frequencies of these variants were compared between patients with acute renal colic and healthy controls. This case–control comparison was intended to characterize differences in variant distribution between the study groups and was not designed to establish genetic susceptibility to acute renal colic.

## 2. Results

### 2.1. Study Population

A total of 250 individuals were enrolled in this prospective case–control study, including 150 patients with acute renal colic and 100 healthy controls. The demographic, laboratory, and clinical characteristics of the study population are presented in Table 1. Patients with acute renal colic presented with severe pain requiring opioid analgesia in the emergency department. Baseline pain intensity, early analgesic response, initial opioid dose, rescue analgesic requirement, stone size, hydronephrosis grade, and routine laboratory parameters were systematically recorded to enable subsequent genotype–phenotype analyses. Genomic DNA of adequate quality was successfully isolated from all participants, and all samples fulfilled the predefined quality control criteria; therefore, no samples were excluded from the pharmacogenetic analyses.

### 2.2. OPRM1 A118G (rs1799971) Polymorphism

Within the patient group, the frequencies of genotypes AA, AG and GG were 97 (64.7%), 44 (29.3%) and 9 (6.0%), respectively. On the other hand, in the control group, these ratios were 64 (64.0%), 32 (32.0%) and 4 (4.0%), respectively. No significant difference was found statistically between the patient and control groups in terms of OPRM1 A118G genotype distributions (*p* = 0.739).

### 2.3. COMT Val158Met (rs4680) Polymorphism

In the patient group, Met/Met (AA) genotype was detected in 24 (16.0%), Val/Met (AG) genotype in 70 (46.7%), and Val/Val (GG) genotype in 56 (37.3%) individuals. In the control group, respectively, 14 (14.0), 51 (51.0), and 35 (35.0) individuals had the corresponding genotypes. No statistically significant difference was observed between the patient and control groups in terms of COMT Val158Met genotype distributions (*p* = 0.786).

### 2.4. ABCB1 C3435T (rs1045642) Polymorphism

Regarding the ABCB1 C3435T polymorphism, in the patient group, CC, CT, and TT genotypes were found in 49 (32.7%), 70 (46.7%), and 31 (20.7) individuals, respectively. In the control group, CC genotype was observed in 37 (37.0%), CT genotype in 52 (52.0%), and TT genotype in 11 (11.0%) individuals. Although the TT genotype is present at a higher rate in the patient group compared to the control group, the overall genotype distribution between the two groups was not statistically significantly different (*p* = 0.134).

### 2.5. Genotype and Allele Distributions of OPRM1, COMT and ABCB1 Polymorphisms

The genotype and allele distributions of the investigated pharmacogenetic polymorphisms are presented in Table 2. The genotype frequencies of OPRM1 A118G, COMT Val158Met, and ABCB1 C3435T polymorphisms did not differ significantly between patients with renal colic and healthy controls (*p* > 0.05 for all comparisons). Similarly, allele frequencies were comparable between the two groups. Although the frequency of the ABCB1 TT genotype was higher among patients with acute renal colic than in controls (20.7% vs. 11.0%), this difference did not reach statistical significance.

### 2.6. Association Analyses Under Different Genetic Models

As a secondary exploratory analysis, differences in the distribution of the investigated polymorphisms between patients with acute renal colic and healthy controls were further evaluated using additive, dominant, and recessive genetic models (Table 3). No statistically significant between-group associations were observed for *OPRM1*, *COMT*, or *ABCB1* under the investigated inheritance models, including the additive per-allele analyses. Under the recessive model, individuals with the *ABCB1* rs1045642 TT genotype had approximately two-fold higher odds of belonging to the acute renal colic group compared with CC/CT carriers (OR = 2.11, 95% CI 1.00–4.42); however, this association was not statistically significant (*p* = 0.057).

The genotype distributions of the investigated polymorphisms were evaluated for Hardy–Weinberg equilibrium (HWE), with primary emphasis on the control group for the secondary case–control analysis (Table 4). In the control group, no significant deviations from HWE were observed for *OPRM1* A118G (rs1799971), *COMT* Val158Met (rs4680), or *ABCB1* C3435T (rs1045642). Given the small number of rare homozygous genotypes, particularly for *OPRM1* rs1799971, HWE was additionally evaluated using an exact test, which similarly showed no evidence of deviation from equilibrium (*OPRM1*: exact *p* = 1.000; *COMT*: exact *p* = 0.675; *ABCB1*: exact *p* = 0.291). Patient-group HWE results are presented descriptively in Table 4. HWE findings were interpreted as a population-level consistency assessment rather than as standalone evidence of genotyping reliability.

### 2.7. Association Between Genotypes and Clinical Outcomes

To investigate whether genetic variation was associated with clinically relevant analgesic outcomes, pain- and treatment-related parameters were compared according to *OPRM1*, *COMT*, and *ABCB1* genotypes. Baseline VAS score, VAS score at 30 min, ΔVAS, initial opioid dose expressed as intravenous morphine-equivalent dose, rescue analgesic requirement, and stone size were evaluated to explore potential genotype–phenotype associations. The findings are presented in Table 5.

The magnitude and precision of the observed genotype–phenotype differences were further examined using effect estimates with 95% CIs (Table 5). For *ABCB1* rs1045642, TT carriers had a higher baseline-adjusted 30 min VAS score than CC carriers (5.26 [95% CI 4.82–5.69] vs. 3.96 [95% CI 3.62–4.31]), corresponding to an adjusted mean difference of approximately 1.30 VAS points. In exploratory unadjusted pairwise comparisons, TT carriers showed a smaller reduction in pain than CC carriers (mean difference in ΔVAS = −0.90, 95% CI −1.46 to −0.34) and received a higher initial weight-based tramadol dose (mean difference = 1.30, 95% CI 0.55–2.05). Rescue fentanyl was required in 41.9% of TT carriers compared with 16.3% of CC carriers, corresponding to an unadjusted OR of 3.70 (95% CI 1.31–10.48). The concordant direction of these effect estimates across pain intensity, pain reduction, initial tramadol requirement, and rescue fentanyl use suggests a potentially clinically relevant pattern; however, these pairwise findings remain exploratory and should be interpreted cautiously, particularly given the relatively small TT subgroup.

**Figure 1:**
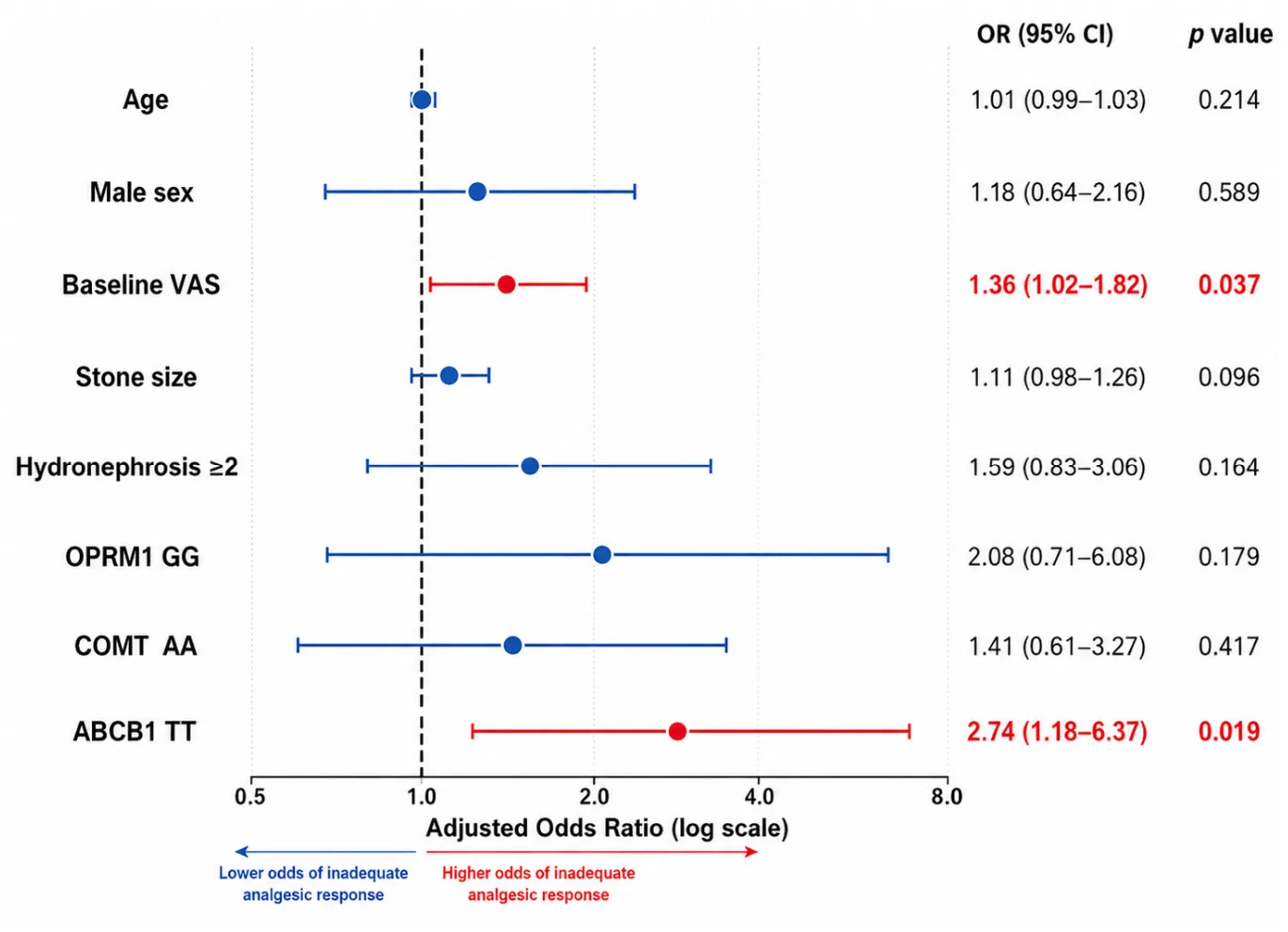
Exploratory multivariable analysis of inadequate early analgesic response in patients with acute renal colic. Inadequate early analgesic response was defined as a VAS score ≥ 4 at 30 min after initial analgesic treatment (corresponding to ≥40 mm on the original 100 mm VAS), whereas a VAS score < 4 was classified as an adequate early analgesic response. The forest plot shows adjusted odds ratios (ORs) with 95% confidence intervals (CIs) from the multivariable logistic regression model including age, sex, baseline VAS score, stone size, hydronephrosis grade, and the investigated genetic variants. Genetic variables were coded under recessive models as follows: *OPRM1* rs1799971, GG versus AA/AG (reference); *COMT* rs4680, AA (Met/Met) versus AG/GG (Val/Met + Val/Val; reference); and *ABCB1* rs1045642, TT versus CC/CT (reference). Baseline VAS score and the *ABCB1* TT genotype were associated with higher odds of inadequate early analgesic response. Given the number of covariates relative to the available outcome events, these multivariable estimates should be interpreted as exploratory and hypothesis-generating rather than as definitive predictive effects.

To further explore whether the investigated genetic variants were associated with early analgesic response after adjustment for potential clinical confounders, an exploratory multivariable logistic regression analysis was performed. The dependent outcome was inadequate early analgesic response, defined as a VAS score ≥ 4 at 30 min after intravenous tramadol administration (corresponding to ≥40 mm on the original 100 mm VAS; versus VAS < 4). The model included age, sex, baseline VAS score, stone size, hydronephrosis grade, *OPRM1* rs1799971, *COMT* rs4680, and *ABCB1* rs1045642. Genetic variables were entered under recessive models: *OPRM1* GG versus AA/AG (reference), *COMT* AA (Met/Met) versus AG/GG (reference), and *ABCB1* TT versus CC/CT (reference). In this exploratory adjusted model, the *ABCB1* TT genotype was associated with higher odds of inadequate early analgesic response (adjusted OR = 2.74, 95% CI 1.18–6.37; *p* = 0.019). Baseline VAS score was also associated with higher odds of inadequate response (adjusted OR = 1.36, 95% CI 1.02–1.82; *p* = 0.037), whereas no statistically significant associations were observed for *OPRM1* or *COMT*. Given the number of covariates relative to the available outcome events and the width of the confidence intervals, these estimates should be interpreted cautiously as exploratory associations rather than definitive predictive effects (Figure 1). Detailed results of the multivariable logistic regression analysis, together with model performance and calibration assessments, are provided in Supplementary Tables S1 and S2 and Supplementary Figure S1.

## 3. Discussion

The present study extends candidate-gene pharmacogenetic research into the acute renal-colic setting, in which rapid pain control is required and opioid therapy may be necessary when first-line treatment is inadequate or contraindicated. The findings should be interpreted as exploratory evidence of interindividual pharmacogenetic variability in opioid response rather than as evidence of genetic susceptibility to renal colic or as a basis for genotype-guided prescribing. Recent pharmacogenetic guidelines and studies have suggested that genotype-informed opioid therapy, particularly using markers such as CYP2D6, may improve analgesic efficacy and optimize opioid prescribing in selected clinical settings [25,26,27]. However, substantial heterogeneity exists across populations, pain conditions, and treatment protocols [28,29], emphasizing the need to interpret candidate-gene associations within their specific clinical context.

The absence of robust adjusted associations for OPRM1 A118G and COMT Val158Met should not necessarily be interpreted as evidence that these pathways are unimportant in opioid analgesia. OPRM1 has been extensively investigated because of its potential influence on μ-opioid receptor function and opioid sensitivity [30,31], whereas COMT may influence catecholamine metabolism, pain perception, and opioid requirements [32,33]. Previous studies have reported heterogeneous effects across populations and clinical settings [28,29,34]. Such variability may reflect differences in phenotype definition, opioid exposure, pain mechanisms, ancestry, treatment protocols, and interactions with additional pharmacokinetic and pharmacodynamic determinants. In the present cohort, the relatively small number of individuals in some genotype strata, particularly the OPRM1 GG subgroup, further limits the precision with which modest genetic effects can be estimated.

The ABCB1 finding warrants consideration from a biological perspective. ABCB1 encodes P-glycoprotein, an ATP-dependent efflux transporter expressed at the blood–brain barrier that can influence central nervous system exposure to certain opioid agents [35,36]. The C3435T (rs1045642) variant is synonymous and should not be interpreted as directly determining P-glycoprotein function; rather, it may represent a marker of ABCB1-related variability, potentially through effects on gene expression or linkage with other functional variants. Thus, the observed association between the TT genotype and early analgesic response is biologically plausible but does not establish a causal mechanism.

Beyond statistical significance, the magnitude and potential clinical relevance of the observed differences should also be considered. The baseline-adjusted mean VAS score at 30 min was 5.26 (95% CI 4.82–5.69) in ABCB1 TT carriers compared with 3.96 (95% CI 3.62–4.31) in CC carriers, corresponding to an approximately 1.3-point difference on the 0–10 VAS. TT carriers also received a higher mean initial weight-based tramadol dose (6.6 vs. 5.3 mg/kg) and more frequently required rescue fentanyl (41.9% vs. 16.3%). The concordant direction of these differences across pain intensity, administered tramadol dose, and rescue fentanyl requirement suggests a potentially clinically relevant pattern rather than an isolated statistical association. However, because no prespecified minimal clinically important difference was defined, the clinical significance of these effect magnitudes cannot be established from the present study alone. Moreover, given the modest sample size, relatively wide confidence intervals, limited number of outcome events, and potential model instability, the ABCB1 association should be regarded as preliminary and hypothesis-generating rather than as evidence of an established predictive biomarker.

From a clinical perspective, these findings should be considered within the established treatment pathway for acute renal colic. The current evidence is insufficient to support routine pharmacogenetic testing before opioid administration or genotype-based dose adjustment. Although genotype-guided approaches are increasingly being evaluated in clinical and pragmatic settings, important challenges remain regarding the evidence base, clinical utility, and implementation of pharmacogenetic testing in routine practice [37,38,39]. Importantly, pharmacogenetic information would not be intended to replace NSAIDs or other established first-line analgesic strategies. Rather, if replicated and clinically validated, genetic information might eventually contribute to individualized opioid selection or dosing specifically among patients who require opioid therapy because first-line treatment is inadequate or contraindicated. In the present treatment pathway, intravenous tramadol was administered as second-line opioid therapy after inadequate response to first-line treatment, whereas intravenous fentanyl was reserved for rescue analgesia in patients with persistent clinically significant pain at 30 min. Future validation should therefore incorporate standardized analgesic protocols, larger prospective cohorts, and broader assessment of relevant pharmacokinetic and pharmacodynamic determinants [40,41].

The present findings also extend our previous pharmacogenetic studies of analgesic response in Turkish patient populations. In those studies, CYP2D6 polymorphisms were associated with tramadol efficacy, CYP2E1 variants with paracetamol response, and CYP2C19 polymorphisms with meperidine analgesia [22,23,24]. Together, these observations support a multifactorial model in which variability in analgesic response may reflect the combined contribution of drug metabolism, receptor signaling, pain modulation, and drug transport rather than the effect of a single polymorphism.

The pharmacogenetic evidence for OPRM1, COMT, and ABCB1 should, however, be distinguished from more clinically established gene–drug relationships in opioid therapy. Associations of these candidate genes with pain perception, opioid requirements, and analgesic response remain heterogeneous across populations and clinical settings and currently do not provide a basis for routine genotype-guided opioid prescribing. In contrast, CYP2D6-dependent metabolism represents a more established pharmacogenetic relationship for opioids such as tramadol and codeine, for which variation in metabolic phenotype can substantially influence active-metabolite formation and consequently analgesic efficacy and toxicity [26,42]. This distinction is particularly relevant to the present study because tramadol constituted the second-line opioid therapy, while CYP2D6 was not assessed. Therefore, unmeasured CYP2D6-dependent variability in tramadol metabolism may have contributed to interindividual differences in analgesic response. The present investigation was intentionally designed as a focused candidate-gene study examining three variants representing complementary mechanisms relevant to opioid response—receptor-mediated pharmacodynamics (OPRM1), pain modulation (COMT), and drug transport (ABCB1)—rather than as comprehensive pharmacogenomic profiling. Additional relevant pharmacogenes, including CYP2D6, were not genotyped within the scope of the present study. Their absence limits the ability to account for genetically determined differences in opioid metabolism and may have contributed to unexplained interindividual variability in analgesic response [43,44].

Importantly, the present study was designed as a preliminary candidate-gene association study rather than as a prediction-model development or validation study. Therefore, it was not designed to determine whether genetic information provides incremental predictive value beyond routinely available clinical variables. Although the ABCB1 TT genotype remained associated with inadequate early analgesic response after adjustment for relevant clinical covariates, this adjusted association does not demonstrate that inclusion of ABCB1 genotype improves individual-level prediction. Given the limited number of outcome events, post hoc comparison of clinical-only and clinical-plus-genetic prediction models could also yield unstable or overfitted estimates. Future adequately powered studies should therefore directly compare clinical and clinical-plus-genetic models and assess discrimination, calibration, and the incremental predictive contribution of genetic information before genotype-informed precision-analgesia approaches are considered for clinical implementation.

Several strengths of the study should be acknowledged. The prospective design enabled systematic collection of clinically relevant analgesic outcomes in a well-defined acute pain setting. Early analgesic response was evaluated using a baseline-adjusted 30 min VAS measure rather than relying exclusively on unadjusted change scores, while ΔVAS was retained as a sensitivity outcome. The stepwise analgesic protocol enabled prospective recording of the administered weight-based tramadol dose and subsequent rescue fentanyl requirement, providing complementary treatment-related outcomes alongside pain intensity. Detailed clinical phenotyping, including stone characteristics and hydronephrosis, further enabled genotype–phenotype associations to be evaluated in the context of clinically relevant factors. Molecular genotyping was supported by predefined quality-control procedures, including repeat genotyping of randomly selected samples, negative and no-template controls, and Sanger sequencing confirmation of representative genotypes. Furthermore, the simultaneous evaluation of candidate variants representing opioid receptor signaling (OPRM1), pain modulation (COMT), and drug transport (ABCB1) provided a biologically complementary framework for exploring interindividual variability in opioid analgesic response. The analyses also incorporated effect estimates with 95% confidence intervals, correction for multiple comparisons, and exploratory multivariable adjustment for relevant demographic and clinical covariates. Nevertheless, these methodological strengths should be considered within the exploratory candidate-gene and observational design and do not establish causal relationships.

Several limitations should also be acknowledged. First, no formal a priori sample-size or power calculation was performed for the genotype–phenotype analyses. Although the study included 150 patients with acute renal colic, some genotype strata were small, particularly the OPRM1 GG subgroup (n = 9), which may have limited statistical power to detect modest genetic effects and resulted in imprecise estimates for rare genotype groups [45]. Accordingly, genotype-specific findings, particularly those involving low-frequency genotypes, should be considered exploratory and interpreted with appropriate caution. Second, only three candidate polymorphisms were investigated. The absence of additional pharmacogenetically important genes, including CYP2D6, may have limited the comprehensive evaluation of genetic influences on opioid response [46,47]. Furthermore, the study was not designed or powered for prediction-model development, and the incremental predictive value of adding genetic information to established clinical predictors was not formally evaluated.

Third, the study population was derived from a single center and exhibited limited demographic diversity, which may restrict the generalizability of the findings [48,49]. Ancestry-informative markers or genome-wide ancestry data were not available to formally assess population stratification. Although patients and controls were recruited from the same geographic catchment area in western Türkiye, residual population substructure cannot be excluded. This limitation is particularly relevant to the secondary case–control analysis because allele frequencies may vary across ancestral and geographic populations; therefore, between-group differences in variant frequencies should be interpreted cautiously. The patient–control comparison should consequently be regarded as a secondary exploratory analysis of variant distribution rather than as evidence of genetic susceptibility to acute renal colic.

In addition, previous opioid exposure, recent use of other analgesics such as paracetamol, concomitant centrally acting medications, pain characteristics, and other clinical factors may have influenced analgesic response. Although regular medication use was recorded, sufficiently detailed and consistently documented exposure data were not available for all participants to permit reliable incorporation of these variables into additional sensitivity or adjusted analyses. Residual confounding related to prior and concomitant medication exposure therefore cannot be excluded [50]. Although analgesic treatment followed a predefined stepwise pathway, tramadol dosing was individualized according to body weight, and rescue fentanyl was administered only to patients with persistent clinically significant pain; therefore, treatment exposure was not completely uniform across participants. Finally, because allele frequencies and pharmacogenetic effects may vary across ancestral and geographic populations, external validation in independent and more diverse cohorts is required before these findings can be generalized [51]. In addition, because this was an observational study, the administered tramadol dose was determined according to body weight and clinical requirements rather than being experimentally assigned. Although genotype information was unavailable to treating physicians and therefore could not directly influence treatment allocation, clinical characteristics associated with analgesic response may also have influenced dosing and rescue-treatment decisions. Consequently, the observed associations between genotype and administered tramadol dose should not be interpreted as evidence that genotype determined opioid dose requirements.

Future studies should evaluate broader pharmacogenetic panels incorporating both pharmacokinetic and pharmacodynamic genes, population-specific allele-frequency information, and pharmacogenetic variability across different classes of analgesic therapies [52,53,54]. Large multicenter prospective cohorts, biobank-based studies, and genome-wide approaches involving diverse populations may further clarify the contribution of common and rare genetic variants to interindividual variability in analgesic response and opioid effectiveness [55,56]. In addition, clinical factors contributing to opioid response, including prior opioid exposure, tolerance, and treatment-related characteristics, should be considered alongside genetic determinants [57]. Such studies should also determine whether genetic information provides incremental predictive value beyond established clinical factors by formally comparing clinical-only and clinical-plus-genetic models and evaluating discrimination, calibration, and clinical utility.

No significant differences in the distributions of the investigated OPRM1, COMT, and ABCB1 variants were observed between patients with acute renal colic and healthy controls. Within the patient group, the ABCB1 rs1045642 TT genotype was independently associated with higher odds of inadequate early analgesic response after adjustment for clinical covariates. Given the exploratory design, modest sample size, and limited number of outcome events, this association should be considered preliminary and requires external validation in larger, independent cohorts before any clinical application can be considered.

## 4. Materials and Methods

### 4.1. Study Design

Both patients and healthy controls were recruited from the same geographic catchment area in Denizli, western Türkiye. Detailed self-reported ancestry, parental geographic origin, ancestry-informative markers, or genome-wide ancestry data were not collected; therefore, genetically inferred ancestry could not be assessed. Recruitment of both groups from the same regional population was intended to reduce major geographic heterogeneity between groups; however, residual population stratification cannot be excluded. The study protocol was approved by the Non-Interventional Clinical Research Ethics Committee of Pamukkale University on 16 June 2026 (Meeting No. 11; official approval document dated 22 June 2026, No. E-60116787-020-889274). A total of 250 individuals were included, comprising 150 consecutive patients diagnosed with acute renal colic and 100 healthy controls. The diagnosis of acute renal colic was established based on clinical presentation and confirmed by ultrasonography and/or non-contrast computed tomography (NCCT). Eligible patients were adults presenting with acute flank pain clinically compatible with renal colic and with a ureteral stone confirmed by ultrasonography and/or non-contrast computed tomography (NCCT). Stone location (proximal, middle, or distal ureter), laterality, maximum stone diameter, and the presence and grade of hydronephrosis were prospectively recorded. Patients were excluded if they had evidence of active urinary tract infection or systemic infection, chronic kidney disease or clinically significant renal impairment unrelated to the acute obstructive episode, chronic pain requiring regular analgesic therapy, chronic or recent opioid use that could affect opioid responsiveness, pregnancy, or another acute condition capable of substantially influencing pain assessment or analgesic requirements. A history of previous or recurrent urolithiasis was recorded where available and was not considered, by itself, an exclusion criterion unless accompanied by factors expected to interfere with assessment of the acute analgesic response. Healthy controls were recruited from the same geographic catchment area as the patient group and had no known history of nephrolithiasis or renal colic. Control eligibility was assessed based on medical history and available clinical records. Individuals with a history of chronic pain requiring regular analgesic treatment, current or recent opioid use, clinically significant renal disease, or other major medical conditions or regular medications considered likely to affect pain perception or analgesic response were excluded. No additional imaging was performed solely for control-group screening; therefore, asymptomatic nephrolithiasis could not be definitively excluded. Healthy controls were included only in the secondary exploratory comparison of genotype and allele frequencies and were not included in the genotype–phenotype analyses of analgesic response.

### 4.2. Analgesic Treatment Protocol

Analgesic treatment was initiated after baseline pain assessment and followed a stepwise approach according to pain severity and clinical response. First-line treatment consisted of a nonsteroidal anti-inflammatory drug (NSAID) administered together with hyoscine butylbromide (Buscopan). In patients with persistent or inadequately controlled pain after first-line treatment, intravenous tramadol was administered as second-line analgesic therapy, with the dose calculated according to body weight (mg/kg) and clinical requirements. The administered tramadol dose was prospectively recorded before assessment of the 30 min analgesic response. Pain intensity was reassessed 30 min after tramadol administration using the Visual Analog Scale (VAS), as described below. Persistent clinically significant pain, defined as a VAS score ≥ 4 at 30 min (corresponding to ≥40 mm on the original 100 mm VAS), prompted rescue analgesia with intravenous fentanyl, with the dose calculated according to body weight (µg/kg). The requirement for rescue fentanyl was prospectively recorded. Genotyping was performed independently of the acute clinical treatment process, and genotype results were not available to the treating physicians at the time of analgesic selection, tramadol dosing, or rescue-treatment decisions. Therefore, treatment allocation and dosing decisions were made without knowledge of genotype.

### 4.3. Clinical Data Collection and Outcome Assessment

Demographic characteristics, including age, sex, body mass index (BMI), smoking status, comorbidities, and regular medication use, were recorded for all participants. For patients with acute renal colic, medication history was reviewed with particular attention to previous opioid exposure and concomitant analgesic or centrally acting medications. However, detailed quantitative information regarding previous opioid exposure, recent paracetamol use, and specific centrally acting medications was not consistently available for all participants and therefore could not be reliably incorporated into additional sensitivity or adjusted analyses. For patients with acute renal colic, additional clinical variables, including stone location, stone diameter, stone laterality, presence and grade of hydronephrosis, and laboratory findings, were prospectively collected. Laboratory investigations included complete blood count, serum creatinine, blood urea nitrogen, C-reactive protein (CRP), and estimated glomerular filtration rate (eGFR).

Pain intensity was assessed using a 100 mm (10 cm) Visual Analog Scale (VAS), anchored by “no pain” at 0 mm and “worst imaginable pain” at 100 mm. For clinical recording and statistical analysis, VAS measurements were expressed on a 0–10 scale by converting the measured distance in millimeters to the corresponding 0–10 value. Pain intensity was assessed immediately before analgesic administration and reassessed 30 min after treatment. The change in pain intensity (ΔVAS) was calculated as the baseline VAS score minus the 30 min VAS score, with higher ΔVAS values indicating greater early pain relief. Persistent clinically significant pain, defined as a VAS score ≥ 4 at 30 min (corresponding to ≥40 mm on the original 100 mm VAS), was considered an inadequate early analgesic response and constituted the criterion for rescue analgesia.

The initial opioid dose, standardized as an intravenous morphine-equivalent dose (mg), requirement for rescue analgesia, emergency department length of stay, and clinical disposition (discharge or hospitalization) were prospectively recorded using standardized case report forms. These clinical outcomes were subsequently evaluated in the genotype–phenotype association analyses.

### 4.4. Sampling

All participants provided approximately 2 mL of peripheral venous blood collected into EDTA-containing tubes. Genomic DNA was isolated using a commercial DNA isolation kit according to the manufacturer’s instructions. The concentration and purity of the isolated DNA were assessed using a NanoDrop spectrophotometer (Thermo Fisher Scientific, Waltham, MA, USA). DNA samples with an A260/A280 ratio of 1.8–2.0 were included in the genotyping analyses.

### 4.5. Genotyping

Three polymorphisms with potential relevance to opioid analgesic response, pain perception, and drug transport were investigated: *OPRM1* A118G (rs1799971), *COMT* Val158Met (rs4680), and *ABCB1* C3435T (rs1045642). Genotyping was performed on genomic DNA obtained from all participants using TaqMan SNP Genotyping Assays (Thermo Fisher Scientific, Waltham, MA, USA) on a LightCycler^®^ 480 Real-Time PCR System (Roche Diagnostics, Mannheim, Germany). PCR amplification was conducted according to the manufacturer’s recommended protocol, and allelic discrimination and genotype assignment were carried out using the instrument’s analysis software.

Genotype calls were accepted based on clear separation of fluorescence clusters in the allelic-discrimination plots. Samples showing absent, weak, or ambiguous allele-discrimination signals were subjected to repeat analysis before final genotype assignment. Complete genotype data were available for all participants for all three investigated polymorphisms, corresponding to an overall call rate of 100% in the final analytical dataset.

Quality-control procedures were implemented to assess genotyping reproducibility. Approximately 10% of the samples were randomly selected for independent repeat genotyping, and the repeated genotype calls were fully concordant with the original assignments. Negative controls and no-template controls were included in each PCR run to monitor potential contamination. In addition, representative samples covering the observed genotype categories were subjected to validation by Sanger sequencing. Following purification of the PCR products, bidirectional sequencing was performed, and the resulting sequences were compared with the corresponding reference sequences to confirm the identified variants.

### 4.6. Statistical Analysis

Statistical analyses were performed using IBM SPSS Statistics version 26.0 (IBM Corp., Armonk, NY, USA). The normality of continuous variables was evaluated using the Shapiro–Wilk test. Normally distributed variables are presented as mean ± standard deviation (SD), whereas non-normally distributed variables are presented as median (interquartile range). Categorical variables are expressed as number (n) and percentage (%).

Demographic and clinical characteristics were compared between patients and controls using the independent-samples *t*-test or Mann–Whitney U test for continuous variables and the Pearson χ^2^ test or Fisher’s exact test for categorical variables. As no individual or frequency matching was performed, all comparisons between patients with acute renal colic and healthy controls were conducted as independent, unmatched-group analyses.

Genotype and allele frequencies were calculated for each polymorphism. For the secondary case–control analysis, Hardy–Weinberg equilibrium (HWE) was evaluated primarily in the control group, as the control population provides the relevant reference for assessing genotype-distribution equilibrium. Given the small numbers of some rare homozygous genotypes, exact HWE testing was used in addition to the χ^2^ goodness-of-fit test where appropriate. HWE was considered a population-level consistency check rather than standalone evidence of genotyping reliability; technical genotyping quality was assessed separately through repeat genotyping, negative and no-template controls, and Sanger sequencing validation. Genotype distributions were evaluated using additive, dominant, and recessive inheritance models.

To investigate genotype–phenotype associations, baseline VAS score, VAS score at 30 min, ΔVAS, initial opioid dose expressed as intravenous morphine-equivalent dose, rescue analgesic requirement, and stone size were evaluated according to genotype. To account for baseline pain intensity, analysis of covariance (ANCOVA) was performed with the 30 min VAS score as the dependent variable, genotype as the fixed factor, and baseline VAS score as the covariate. Separate ANCOVA models were performed for *OPRM1* rs1799971, *COMT* rs4680, and *ABCB1* rs1045642. Adjusted estimated marginal means with 95% confidence intervals (CIs) were calculated. ΔVAS, defined as the difference between baseline and 30 min VAS scores, was retained as a sensitivity analysis. Other continuous clinical outcomes were compared among genotype groups using one-way analysis of variance (ANOVA) or the Kruskal–Wallis test, as appropriate. Categorical clinical outcomes were compared using the χ^2^ test or Fisher’s exact test.

For the revised analytical framework, the baseline-adjusted 30 min VAS score was treated as the principal clinical measure of early analgesic response, whereas ΔVAS was retained as a sensitivity outcome. Other genotype–phenotype comparisons, including initial opioid dose, rescue analgesic requirement, stone size, and analyses under alternative inheritance models, were considered exploratory. Because no formal hierarchical multiplicity-testing strategy had been specified, these analyses were not interpreted as independent confirmatory tests. To account for multiple testing across the 18 genotype–phenotype comparisons presented in Table 5, the Benjamini–Hochberg false discovery rate (FDR) procedure was applied, and both nominal and FDR-adjusted *p* values were reported. Nominal *p* values < 0.05 were used for descriptive inference, whereas FDR-adjusted *p* values < 0.05 were considered to indicate associations that remained statistically significant after multiplicity correction.

To further explore associations with inadequate early analgesic response, defined as a VAS score ≥ 4 at 30 min after initial analgesic treatment (corresponding to ≥40 mm on the original 100 mm VAS; versus VAS < 4), an exploratory multivariable logistic regression analysis was performed. The model included age, sex, baseline VAS score, stone size, hydronephrosis grade, and the investigated genetic polymorphisms. For this analysis, genetic variables were entered using recessive coding: *OPRM1* rs1799971 as GG versus AA/AG (reference), *COMT* rs4680 as AA (Met/Met) versus AG/GG (Val/Met + Val/Val; reference), and *ABCB1* rs1045642 as TT versus CC/CT (reference). Recessive coding was used to provide clinically interpretable binary genetic contrasts and to limit model complexity by avoiding separate parameter estimates for all three genotype categories. Adjusted odds ratios (ORs) with 95% CIs were calculated. Given the number of covariates relative to the available outcome events, the multivariable model was considered exploratory, and its estimates were interpreted cautiously rather than as definitive predictive effects.

No formal a priori sample-size or power calculation was performed for the genotype–phenotype analyses. Therefore, these analyses were considered exploratory, particularly for low-frequency genotype strata.

## Figures and Tables

**Table 1:** Demographic and clinical characteristics of patients with acute renal colic and healthy controls.

Variable	Patients with Acute Renal Colic (n = 150)	Healthy Controls (n = 100)	*p* Value
Age, years	37.0 (29.0–49.0)	45.0 (32.0–65.0)	0.002
Male sex, n (%)	91 (60.7)	46 (46.0)	0.031
Creatinine, mg/dL	0.87 (0.72–1.03)	0.81 (0.66–0.93)	0.012
Baseline VAS score	8.3 ± 1.1	—	—
VAS score at 30 min	4.7 ± 1.8	—	—
ΔVAS (baseline–30 min)	3.6 ± 1.7	—	—
Initial tramadol dose	5.8 ± 2.1 ^†^	—	—
Rescue fentanyl required, n (%)	38 (25.3)	—	—
Stone size, mm	**4.0 (3.0–7.0)**	—	—
Hydronephrosis grade 0/1/2/3, n	64/49/33/4	—	—
Hematuria, n (%)	138 (92.0)	—	—
Costovertebral angle tenderness, n (%)	133 (88.7)	—	—

Data are presented as median (interquartile range [IQR]), mean ± standard deviation (SD), or n (%), as appropriate. The distribution of continuous variables was assessed using the Shapiro–Wilk test together with visual inspection of the data distribution. Non-normally distributed continuous variables were compared using the Mann–Whitney U test, whereas categorical variables were compared using the Pearson χ^2^ test or Fisher’s exact test, as appropriate. Patient-specific clinical and analgesic variables were not applicable to healthy controls. Abbreviations: VAS, Visual Analog Scale; ΔVAS, change in VAS score from baseline to 30 min. ^†^ Values are expressed as mean ± standard deviation.

**Table 2:** Genotype and allele distributions of OPRM1, COMT and ABCB1 polymorphisms.

Gene	Genotype	Patients n (%)	Controls n (%)	*p* Value
*OPRM1* (rs1799971)	AA	97 (64.7)	64 (64.0)	
	AG	44 (29.3)	32 (32.0)	
	GG	9 (6.0)	4 (4.0)	0.739
*COMT* (rs4680)	AA (Met/Met)	24 (16.0)	14 (14.0)	
	AG (Val/Met)	70 (46.7)	51 (51.0)	
	GG (Val/Val)	56 (37.3)	35 (35.0)	0.786
*ABCB1* (rs1045642)	CC	49 (32.7)	37 (37.0)	
	CT	70 (46.7)	52 (52.0)	
	TT	31 (20.7)	11 (11.0)	0.134

**Table 3:** Association analyses under dominant and recessive genetic models.

Gene	Genetic Model	Comparison Category	Reference Category	OR (95% CI)	*p* Value
*OPRM1*	Dominant	AG + GG	AA	0.97 (0.57–1.65)	1.000
*OPRM1*	Recessive	GG	AA + AG	1.53 (0.46–5.12)	0.572
*COMT*	Dominant	AG + GG	AA	0.85 (0.42–1.75)	0.722
*COMT*	Recessive	GG	AA + AG	1.11 (0.65–1.88)	0.789
*ABCB1*	Dominant	CT + TT	CC	1.21 (0.71–2.06)	0.499
*ABCB1*	Recessive	TT	CC + CT	2.11 (1.00–4.42)	0.057

ORs represent the odds of belonging to the acute renal colic patient group relative to healthy controls for the comparison category versus the explicitly specified reference category. ORs > 1 indicate higher odds of belonging to the patient group for the comparison category relative to the reference category. Genotype counts and allele frequencies for patients and controls are presented in Table 2. Abbreviations: OR, odds ratio; CI, confidence interval.

**Table 4:** Hardy–Weinberg equilibrium analysis.

Gene	Group	χ^2^	*p* (χ^2^ HWE)	Exact HWE *p*
*OPRM1*	Controls	0.000	1.000	1.000
	Patients	1.668	0.197	0.212
*COMT*	Controls	0.450	0.502	0.675
	Patients	0.074	0.786	0.864
*ABCB1*	Controls	1.332	0.248	0.291
	Patients	0.422	0.516	0.511

Hardy–Weinberg equilibrium (HWE) was evaluated separately in healthy controls and patients with acute renal colic using both the χ^2^ test and an exact HWE test. For the case–control genetic association analysis, HWE results in the healthy control group were considered the primary assessment for identifying potential genotyping- or population-related deviations, whereas patient-group results are presented for completeness. No investigated variant showed evidence of deviation from HWE in the control group (all exact HWE *p* > 0.05). Exact HWE *p* values were used as the primary HWE assessment.

**Table 5:** Associations of *OPRM1*, *COMT*, and *ABCB1* genotypes with pain intensity and analgesic outcomes in patients with acute renal colic.

**Panel A. Continuous Analgesic Outcomes**					
**Gene**	**Genotype**	**Adjusted VAS at 30 min, Mean (95% CI)**	**ΔVAS, Mean (95% CI)**		**Initial Tramadol Dose, Mean (95% CI)**
*OPRM1* rs1799971	AA (n = 97)	4.31 (4.07–4.55)	3.90 (3.64–4.16)		5.40 (5.10–5.70)
	AG (n = 44)	4.78 (4.42–5.13)	3.50 (3.07–3.93)		5.90 (5.35–6.45)
	GG (n = 9)	5.52 (4.73–6.31)	2.90 (1.98–3.82)		6.80 (5.49–8.11)
Overall nominal *p*		0.004	0.043		0.020
FDR-adjusted *p*		0.018	0.086		0.051
*COMT* rs4680	AA (Met/Met) (n = 24)	5.40 (4.91–5.89)	3.40 (2.89–3.91)		6.00 (5.32–6.68)
	AG (Val/Met) (n = 70)	4.49 (4.21–4.78)	3.70 (3.37–4.03)		5.70 (5.29–6.11)
	GG (Val/Val) (n = 56)	4.47 (4.16–4.79)	3.90 (3.55–4.25)		5.40 (4.97–5.83)
Overall nominal *p*		0.004	0.302		0.300
FDR-adjusted *p*		0.018	0.418		0.418
*ABCB1* rs1045642	CC (n = 49)	3.96 (3.62–4.31)	4.00 (3.63–4.37)		5.30 (4.90–5.70)
	CT (n = 70)	4.40 (4.12–4.69)	3.70 (3.37–4.03)		5.80 (5.39–6.21)
	TT (n = 31)	5.26 (4.82–5.69)	3.10 (2.66–3.54)		6.60 (5.94–7.26)
Overall nominal *p*		<0.001	0.014		0.003
FDR-adjusted *p*		≤0.018 ^†^	0.042		0.018
**Panel B. Categorical Analgesic Outcome: Rescue Fentanyl Requirement**					
**Gene**	**Genotype**	**Rescue Fentanyl, n/N (%)**	**Comparison**	**OR (95% CI)**	***p* Value**
*OPRM1* rs1799971	AA	20/97 (20.6)	Reference	1.00	—
	AG	12/44 (27.3)	AG vs. AA	1.44 (0.63–3.30)	0.383
	GG	6/9 (66.7)	GG vs. AA	7.70 (1.77–33.51)	0.007
Overall genotype effect					Nominal *p* = 0.009; FDR *p* = 0.032
*COMT* rs4680	GG (Val/Val)	12/56 (21.4)	Reference	1.00	—
	AG (Val/Met)	18/70 (25.7)	AG vs. GG	1.27 (0.55–2.92)	0.575
	AA (Met/Met)	8/24 (33.3)	AA vs. GG	1.83 (0.63–5.30)	0.263
Overall genotype effect					Nominal *p* = 0.530; FDR *p* = 0.636
*ABCB1* rs1045642	CC	8/49 (16.3)	Reference	1.00	—
	CT	17/70 (24.3)	CT vs. CC	1.64 (0.65–4.18)	0.297
	TT	13/31 (41.9)	TT vs. CC	3.70 (1.31–10.48)	0.014
Overall genotype effect					Nominal *p* = 0.036; FDR *p* = 0.081

**Panel A.** Data for continuous outcomes are presented as adjusted estimated marginal means with 95% confidence intervals (CIs) or unadjusted means with 95% CIs, as appropriate. Thirty-minute VAS scores were analyzed using analysis of covariance (ANCOVA), with genotype as the fixed factor and baseline VAS score as the covariate; therefore, baseline-adjusted estimated marginal means with 95% CIs are reported. ΔVAS (baseline VAS minus 30 min VAS) and initial weight-based tramadol dose are presented as unadjusted means with 95% CIs and were compared across genotype groups using one-way analysis of variance (ANOVA). Overall nominal *p* values represent omnibus genotype effects. To account for multiple genotype–phenotype comparisons, nominal *p* values were adjusted using the Benjamini–Hochberg false discovery rate (FDR) procedure, and both nominal and FDR-adjusted *p* values are reported. FDR-adjusted *p* values < 0.05 were considered statistically significant after multiplicity correction. Abbreviations: VAS, Visual Analog Scale; ΔVAS, change in VAS score from baseline to 30 min; CI, confidence interval; FDR, false discovery rate. Because multiple genotype–phenotype comparisons were performed, the 18 nominal *p* values presented in Table 5 were additionally subjected to Benjamini–Hochberg false discovery rate (FDR) correction. After multiplicity adjustment, the genotype-related differences in baseline-adjusted 30 min VAS remained statistically significant for OPRM1, COMT, and ABCB1. The genotype-related difference in rescue fentanyl requirement for OPRM1 and the differences in ΔVAS and initial weight-based tramadol dose for ABCB1 also remained statistically significant after FDR correction. In contrast, the nominal differences in ΔVAS and initial weight-based tramadol dose for OPRM1 and in rescue fentanyl requirement for ABCB1 did not remain statistically significant after multiplicity adjustment. **Panel B.** Rescue fentanyl requirement is presented as n/N (%). Genotype-specific associations with rescue fentanyl requirement were evaluated using pairwise odds ratios (ORs) with 95% confidence intervals (CIs), calculated relative to the explicitly specified reference genotype for each gene. ORs > 1 indicate higher odds of requiring rescue fentanyl compared with the reference genotype. Pairwise *p* values correspond to the reported genotype contrasts. Overall nominal *p* values represent omnibus comparisons of rescue fentanyl requirement across the three genotype groups using the Pearson χ^2^ test. To account for multiple genotype–phenotype comparisons, the overall nominal *p* values were included in the Benjamini–Hochberg false discovery rate (FDR) correction, and the corresponding FDR-adjusted *p* values are reported. FDR-adjusted *p* values < 0.05 were considered statistically significant after multiplicity correction. Pairwise OR analyses were exploratory and were not separately subjected to FDR correction. Abbreviations: OR, odds ratio; CI, confidence interval; FDR, false discovery rate. ^†^ FDR-adjusted *p* ≤ 0.018 was defined as the threshold for statistical significance after false discovery rate (FDR) correction for multiple comparisons.

## Data Availability

The original contributions presented in this study are included in the article. Further inquiries can be directed to the corresponding author.

## Supplementary Materials

The following supporting information can be downloaded at: https://www.mdpi.com/article/10.3390/ph19091343/s1, Supplementary Table S1: Apparent performance and diagnostic measures of the exploratory multivariable logistic regression model for inadequate early analgesic response; Supplementary Figure S1: Calibration plot for the exploratory multivariable logistic regression model; Supplementary Table S2: Exploratory multivariable logistic regression analysis of inadequate early analgesic response.

## Abbreviations

The following abbreviations are used in this manuscript:

ABCB1	ATP-binding cassette subfamily B member 1
BMI	Body mass index
CI	Confidence interval
COMT	Catechol-O-methyltransferase
CRP	C-reactive protein
DNA	Deoxyribonucleic acid
eGFR	Estimated glomerular filtration rate
HWE	Hardy–Weinberg equilibrium
NSAIDs	Nonsteroidal anti-inflammatory drugs
OPRM1	μ-Opioid receptor 1
OR	Odds ratio
PCR	Polymerase chain reaction
P-gp	P-glycoprotein
Real-Time PCR	Real-time polymerase chain reaction
SNP	Single nucleotide polymorphism
