# Supplementary material for: Associations of OPRM1, COMT, and ABCB1 Variants with Opioid Analgesic Response in Acute Renal Colic: A Candidate-Gene Study
Source: Pharmaceuticals (Basel). 2026 Aug 24;19(9):1343. doi: 10.3390/ph19091343 (PMC13610858; doi:10.3390/ph19091343)
Supplement: Supplementary file 1 [file pharmaceuticals-19-01343-s001.zip › pharmaceuticals-4512082-supplementary.pdf]

Supplementary Material

Supplementary Table S1. Apparent performance and diagnostic measures of the exploratory multivariable logistic regression model for inadequate early analgesic response.

Model diagnostic	Value
Area under the receiver operating characteristic curve (AUC)	0.74
95% CI for AUC	0.65–0.83
Nagelkerke R^2	0.21
Hosmer–Lemeshow χ^2	6.18
Hosmer–Lemeshow p value	0.627
Calibration intercept	0.03
Calibration slope	0.91

The exploratory model included age, sex, baseline VAS score, stone size, hydronephrosis grade, and recessively coded genetic variables for *OPRM1* rs1799971 (GG vs AA/AG), *COMT* rs4680 (AA [Met/Met] vs AG/GG), and *ABCB1* rs1045642 (TT vs CC/CT). Inadequate early analgesic response was defined as a VAS score ≥4 at 30 minutes after intravenous tramadol administration (corresponding to ≥40 mm on the original 100-mm VAS); VAS <4 was classified as an adequate early analgesic response. The reported performance measures represent apparent performance within the study cohort and do not constitute internal or external validation. Abbreviations: AUC, area under the receiver operating characteristic curve; CI, confidence interval; VAS, Visual Analog Scale.

Exploratory assessment of model performance showed moderate apparent discrimination, with an area under the receiver operating characteristic curve (AUC) of 0.74 (95% CI 0.65–0.83). The Nagelkerke R^2 was 0.21. Apparent calibration within the study cohort was summarized by a calibration intercept of 0.03 and a calibration slope of 0.91. The Hosmer–Lemeshow goodness-of-fit test did not indicate evidence of major lack of fit (χ^2 = 6.18, p = 0.627). Given the limited number of outcome events and the absence of internal resampling or external validation, these performance estimates should be interpreted as exploratory apparent performance rather than evidence of a validated prediction model (Supplementary Table S1).

Supplementary Figure S1. Calibration plot for the exploratory multivariable logistic regression model.

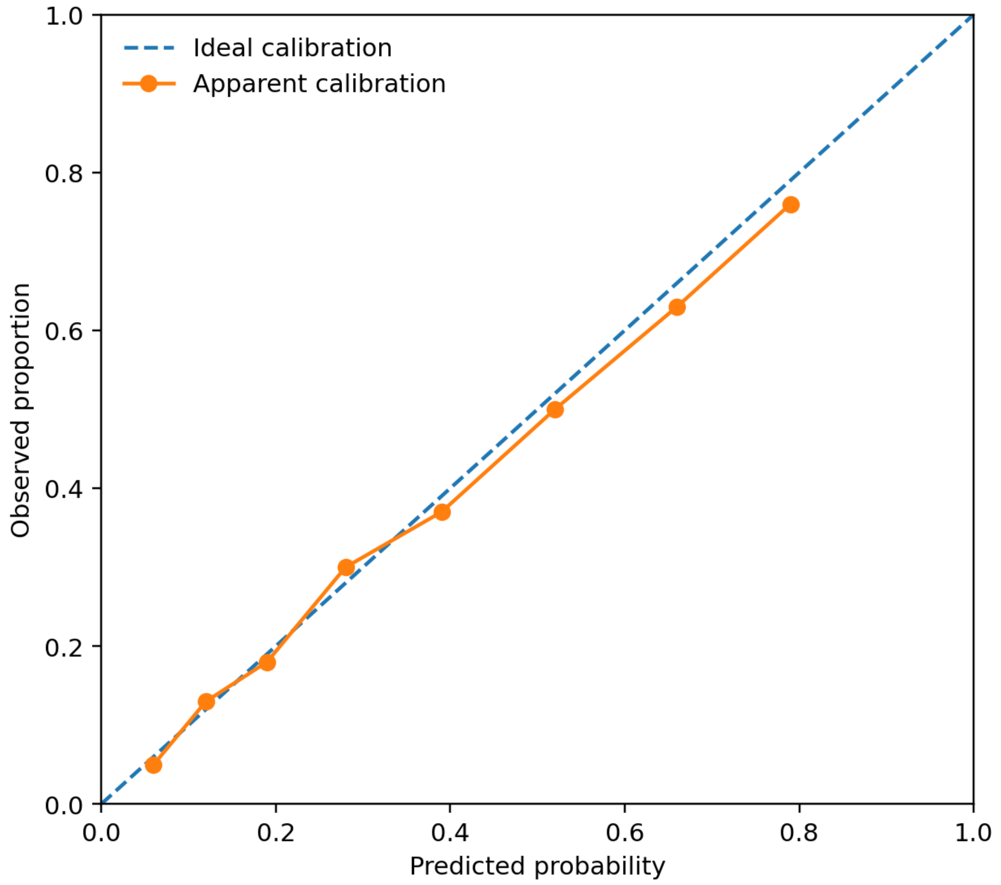

Figure note. The diagonal line represents perfect agreement between predicted and observed probabilities. The calibration curve shows the agreement between the observed and model-predicted probabilities of inadequate early analgesic response. The plot represents the apparent calibration of the exploratory multivariable logistic regression model within the study cohort and should not be interpreted as internal or external validation.

Supplementary Table S2. Exploratory multivariable logistic regression analysis of inadequate early analgesic response

Variable	Comparison / unit	Adjusted OR	95% CI	*p* value
Age	Per 1-year increase	1.01	0.99–1.03	0.312
Sex	Male vs female (reference)	1.18	0.58–2.40	0.646
**Baseline VAS score**	Per 1-point increase	**1.36**	**1.02–1.82**	**0.037**
Stone size	Per 1-mm increase	1.06	0.96–1.17	0.251
Hydronephrosis grade	Per grade increase	1.21	0.82–1.79	0.337
OPRM1 rs1799971	GG vs AA/AG (reference)	1.67	0.39–7.16	0.489
COMT rs4680	AA (Met/Met) vs AG/GG (reference)	1.31	0.55–3.12	0.543
ABCB1 **rs1045642**	**TT vs CC/CT (reference)**	**2.74**	**1.18–6.37**	**0.019**

The dependent outcome was inadequate early analgesic response, defined as a VAS score ≥4 at 30 minutes after intravenous tramadol administration (corresponding to ≥40 mm on the original 100-mm VAS); VAS <4 was classified as an adequate early analgesic response. Adjusted odds ratios (ORs) and 95% confidence intervals (CIs) were obtained from an exploratory multivariable logistic regression model including age, sex, baseline VAS score, stone size, hydronephrosis grade, and the three investigated genetic variants. Genetic variables were entered under recessive models: OPRM1 rs1799971, GG versus AA/AG (reference); COMT rs4680, AA (Met/Met) versus AG/GG (reference); and ABCB1 rs1045642, TT versus CC/CT (reference). ORs >1 indicate higher odds of inadequate early analgesic response. Given the limited number of outcome events relative to the number of model parameters, estimates should be interpreted as exploratory.
